# Do Bodybuilders Use Evidence-Based Nutrition Strategies to Manipulate Physique?

**DOI:** 10.3390/sports5040076

**Published:** 2017-09-29

**Authors:** Lachlan Mitchell, Daniel Hackett, Janelle Gifford, Frederico Estermann, Helen O’Connor

**Affiliations:** 1 Discipline of Exercise and Sport Science, Faculty of Health Sciences, University of Sydney, 75 East Street, Lidcombe NSW 2141, Australia; daniel.hackett@sydney.edu.au (D.H.); janelle.gifford@sydney.edu.au (J.G.); fest5296@uni.sydney.edu.au (F.E.); helen.oconnor@sydney.edu.au (H.O.C.); 2 Charles Perkins Centre, University of Sydney, D17 John Hopkins Drive, Camperdown NSW 2050, Australia

**Keywords:** protein, peak week, refeed day, supplement

## Abstract

Competitive bodybuilders undergo strict dietary and training practices to achieve an extremely lean and muscular physique. The purpose of this study was to identify and describe different dietary strategies used by bodybuilders, their rationale, and the sources of information from which these strategies are gathered. In-depth interviews were conducted with seven experienced (10.4 ± 3.4 years bodybuilding experience), male, natural bodybuilders. Participants were asked about training, dietary and supplement practices, and information resources for bodybuilding strategies. Interviews were transcribed verbatim and analyzed using qualitative content analysis. During the off-season, energy intake was higher and less restricted than during the in-season to aid in muscle hypertrophy. There was a focus on high protein intake with adequate carbohydrate to permit high training loads. To create an energy deficit and loss of fat mass, energy intake was gradually and progressively reduced during the in-season via a reduction in carbohydrate and fat intake. The rationale for weekly higher carbohydrate refeed days was to offset declines in metabolic rate and fatigue, while in the final “peak week” before competition, the reasoning for fluid and sodium manipulation and carbohydrate loading was to enhance the appearance of leanness and vascularity. Other bodybuilders, coaches and the internet were significant sources of information. Despite the common perception of extreme, non-evidence-based regimens, these bodybuilders reported predominantly using strategies which are recognized as evidence-based, developed over many years of experience. Additionally, novel strategies such as weekly refeed days to enhance fat loss, and sodium and fluid manipulation, warrant further investigation to evaluate their efficacy and safety.

## 1. Introduction

Competitive bodybuilders undergo strict dietary and training practices to achieve an extremely lean, muscular and symmetrical physique [1]. Along with resistance and aerobic exercise [2], targeted energy and macronutrient intakes are followed to accumulate muscle mass in the off-season, and reduce fat mass in the in-season [1]. However the specific dietary strategies employed by bodybuilders and their underpinning rationale remain poorly understood.

Contemporary literature examining the dietary intakes of bodybuilders is limited [1], and given the unique nature of competitive bodybuilding, it may be inappropriate to draw dietary parallels from other sports. Although bodybuilders have been reported to follow extreme, non-evidence-based approaches, several dietary strategies developed in bodybuilding have recently been scientifically validated, such as frequent dosing of protein [3], and intake of protein around training [4]. Identifying the dietary strategies of modern bodybuilders, and exploring their underpinning rationale, will provide exercise, sport and nutrition practitioners with an understanding of current bodybuilding methods and insights to assist with negotiating practical and effective ways to work towards bodybuilding goals. Furthermore, identifying such strategies will also generate hypotheses for future research.

In-depth interviews allow a deep exploration of the discussed topic, enable the researchers to enter new areas and produce rich data, with an additional benefit of uncovering practices that had not been anticipated [5,6]. The purpose of this study was to use in-depth interviews to identify and describe different dietary strategies used by male, natural bodybuilders, their rationale, and the sources of education from which these strategies are gathered.

## 2. Materials and Methods

Participants were purposively selected by the research team based on expertise and experience in competitive bodybuilding. To recruit participants, experienced bodybuilders known to the researchers from previous studies were invited to participate. Adverts were placed on the website and social media page of Australasian Natural Bodybuilding (ANB), and distributed at the ANB national titles in October 2015. To be included, participants needed to be male, natural (drug-free) bodybuilders, aged 18 years and older, with five or more years of bodybuilding experience. Participants were required to have competed in the bodybuilding category at national or international level contests of drug-tested federations.

### 2.1. Procedures

The interviews were conducted by three members of the research team between March 2015 and February 2016. Interviews (78–124 min) were held by telephone or Skype. The combined duration of all interviews was 11 h. Interviews captured participant demographic characteristics including age, years of bodybuilding experience, number of previous competitions, and competition success. Participants were asked about their training, dietary, supplement and competition preparation practices, the rationale behind these practices, and where they obtained information about nutrition and training. By the end of the last interview, no new major themes were emerging. Saturation was confirmed following coding of the data, therefore the decision was made to cease further data collection.

### 2.2. Analysis

All interviews were digitally recorded and transcribed by a commercial transcription service (waywithwords.com). Transcripts were returned to participants for verification and correction to ensure the transcription correctly reflected the content of their interview. One participant returned the transcript with minor emendations, which was included in the analysis. Notes were taken during all interviews and used to clarify transcription errors, and to confirm the meaning of spoken phrases during the coding process. To protect the identity of the participants, a pseudonym was used in the final transcripts. All interviews were conducted prior to thematic analysis via qualitative content analysis using qualitative data analysis software (NVivo version 10.0, QSR International PTY Ltd., Doncaster, Australia, 2012). Coding was undertaken by one researcher (LM) with assistance from a second (FE) and overseen by a third researcher experienced in qualitative research (JG), who reviewed any queries. As coding of data proceeded, underlying themes emerged as participants discussed topics introduced by the interviewers, and was not constrained by the original structure of the interview. Identification of themes recurring through and across interviews was achieved through a process of reading, coding, code category refinement, rereading and code checking, and analysis of developing concepts. A coding journal with an audit trail of changes in coding and code refinement was maintained by the primary coder (LM) to maintain transparency of the qualitative analysis process.

Counts of coded talk were available from the analysis software by grouping for diet, training, supplements, and information and education. Counts within themes could have more than one section of speech by the same participant. To avoid researcher bias during the data interpretation process based on preconceived ideas of bodybuilding practices, identified themes were sent to participants, who confirmed correct interpretation.

Ethical approval was received from the University of Sydney Human Ethics Committee. Written informed consent was provided by all participants. Participation was voluntary and identity of participants and confidentiality of their responses was ensured.

## 3. Results 

A total of seven bodybuilders (10.4 ± 3.4 years bodybuilding experience) meeting inclusion criteria responded to advertisements and consented to participate. Participant characteristics are summarized in Table 1. Four participants had competed at national, and three at international level. Two participants had competed professionally, with an additional one participant eligible to compete professionally. Example quotes are presented in Table 2. Selected quotes were representative of themes identified during interviews.

### 3.1. Diet

#### 3.1.1. Off-Season 

All participants consumed four to six meals per day, with a targeted energy and macronutrient intake aimed to support muscular hypertrophy, “I’ve got 250 [g/day] protein, and at the moment I’ll divvy my fats and carbs up, so 250 [g] protein, 680 [g] carb and about 100, 110 [g] on fats, somewhere there,” (Keith). Each meal featured a large serving of a high protein food and a large serving of vegetables, “In the morning I start off with 100 g of oats and six whole eggs. That’s at around about 7:00 a.m. At 9:30 a.m. will be 200 g of salmon and 200 g of green veg,” (Luke). The off-season diet contained a wide variety of foods, including processed foods such as ice cream, and was less regimented than the in-season.

#### 3.1.2. In-Season 

While the pattern and style of the diet was similar to the off-season, the in-season intake was more structured, “It’s more structured, it’s perfect” (Kyle), and usually carefully measured, “I will split a grain of rice, if it made it hit exactly the grammage (sic) I want,” (Keith). Serving sizes were also reduced as competition approached.

**Figure 1:**
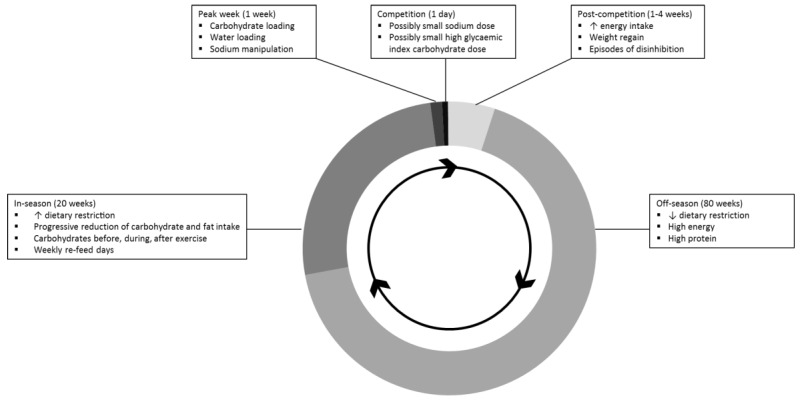
Doughnut chart representation of the stages of bodybuilding preparation, including key dietary strategies used, as reported by seven male, competitive natural bodybuilders participating in in-depth interviews. Duration of stages are approximate and vary between bodybuilders.

Progressive reductions in carbohydrate and fat intake were used to create then maintain an energy deficit to elicit fat loss (Figure 1). Protein intake remained similar to the off-season to prevent loss of lean mass. Carbohydrate intake was carefully timed around exercise (pre-, during and post-training) to ensure training was optimized.

#### 3.1.3. Refeed Days

Refeed days were commonly used during the in-season and primarily aimed to increase energy intake through elevated carbohydrate consumption. Participants discussed positive outcomes including increased glycogen stores which aid training performance, mental recovery, and prevention of further adaptive downgrades in energy expenditure, stimulating weight loss. One participant described it as a “metabolic jumpstart” (Oliver). Compared to preparations without refeed days, participants discussed consuming more total energy, over a shorter preparation, achieving better fat loss and muscle retention using weekly refeed days.

#### 3.1.4. Peak Week

The week prior to the contest was defined as a “peak week”, where particular short-term strategies were used to achieve the leanest possible appearance. Six participants used a modified carbohydrate loading regimen (tapered training and increased carbohydrate intake) [7] in order to increase glycogen and theoretically increase muscle volume. Four participants had previously used the classic loading method, which involved a three-day glycogen depletion and then super-compensation [8], however found this did not produce significant changes in appearance, describing this method as, “stressful,” (Ben) “mentally that would be really bad,” (Kyle) and, “you’re just a wreck” (Luke).

All seven participants discussed the practice of water loading and cutting during peak week. Users of this strategy consumed more than 10 L of water per day early in the week, then reduced water intake each day leading into the contest. The rationale for this strategy was to increase fluid excretion and to “go after subcutaneous water” (Will), which would purportedly provide a leaner, more vascular appearance. Results were not effective enough for these participants to warrant continuation of this strategy in subsequent competition preparations. Other participants commented that the idea of water loading and cutting does not make sense physiologically: “muscle is about 70% water. If you were dehydrated, the muscles are going to look smaller as well,” (Harry).

Sodium manipulation was another strategy used during the peak week to reduce body water and produce a leaner appearance. Three participants discussed previously using this strategy, whereby sodium intake was greatly increased for three days, followed by a complete restriction of salt for three days. However, they each reported that the results were inconsistent, and discontinued the strategy.

#### 3.1.5. Competition Day

Six participants discussed diet strategies used on the day of competition. Two consumed sodium prior to posing on stage to get a greater “pump”. Small doses of high glycemic index carbohydrates were consumed by two participants. One justified this by saying, “That was just to keep you ticking, when you’re feeling that depleted, just to keep you propped up,” (Oliver) while the other participant commented, “That’s for sugars, to get the pump” (Kyle). Two participants did not change from their usual intake on competition day.

#### 3.1.6. Post-Competition

Participants reported the post-competition diet was more relaxed (*n* = 5), and included some “treat” foods not consumed during the in-season. Overindulgence and the experience of feeling physically sick from the change in diet pattern (*n* = 2) was reported. Weight regain was common and could be substantial (8–10 kg over three weeks in one case). Limited time off dieting was reported by three participants to avoid detrimental physique changes. Participants reported negative changes in physique were common post-competition.

### 3.2. Supplements 

All participants used one or more dietary supplements. In total, 18 different supplement types were mentioned. Creatine (3–15 g/d) was used by all participants with doses consumed either pre- or post-workout, with a meal, or a combination of these. Protein powders were also used by all participants either as a post-training supplement (*n* = 4) or as a source of protein during meals (*n* = 4). “Preworkout” supplements designed to stimulate enhanced training was discussed by four participants, one of which used these for their caffeine content, while the others discontinued use due to side effects (insomnia, increased and variable heart rate, and increased respiratory rate). Participants reported these experiences were: “absolutely horrible” (Ben), “I just can’t stand it, frankly,” (Will) and “it’s counterproductive, so I don’t use it” (Will). Other supplements more commonly used were fish oil (four participants), glutamine (three participants) and testosterone boosters (three participants).

### 3.3. Sources of Education

The most commonly reported sources of education were the internet, including bodybuilding, strength and conditioning websites and forums (*n* = 5), successful bodybuilders (*n* = 4), and bodybuilding coaches (*n* = 4). The quality of information available on the internet was considered to be both reputable and non-reputable. Concerns were raised by two participants regarding information on social media, where images and information may be unrealistic and deceptive, and potentially damaging for novices. Bodybuilding coaches were also commonly used, although one participant commented on the varying levels of coach knowledge, with many relying on their own competition experience.

## 4. Discussion

The rationale and use of several key dietary strategies emerged from this study, including regular doses of protein throughout the day to maximize accrual and maintenance of lean mass, and utilizing carbohydrate foods as a fuel source pre-, during and post-exercise. Weekly refeed days were implemented during the in-season, to provide both a psychological rest and reportedly assist with fat loss. During the peak week, bodybuilders followed extreme strategies including water and sodium manipulation in an attempt to achieve the leanest physique.

Throughout both the off-season and in-season, participants reported consuming large, frequent servings of protein to build and maintain muscle mass, which is empirically supported in the research literature [3]. The optimal dose to achieve this maximal muscle protein synthesis is accepted to be 20–30 g of high quality protein [3,9], with studies supporting that protein ingestion above this dose is oxidized [9]. Recent findings suggest the amount of muscle mass trained may be a determinant of protein requirements post-exercise. Greater myofibrillar fractional synthetic rate was achieved with a 40 versus 20 g dose of whey protein following whole-body resistance exercise [10]. Therefore, a dose up to 40 g may produce increased protein synthesis following resistance exercise incorporating large amounts of muscle, such as those followed by bodybuilders.

The high-protein meals consumed by participants in this study likely exceeded the 20–40 g dose for maximal protein synthesis, potentially resulting in increased protein oxidation. However, the anabolic response to protein ingestion is a combination of protein synthesis and breakdown [11]. Greater protein net balance has been produced from a 70 g versus 40 g dose of protein, primarily by decreasing the rate of protein breakdown [11]. Therefore, the frequent, higher-dosed protein meals consumed by bodybuilders may not only assist in supporting protein synthesis, but also in reducing protein degradation during heavy resistance training. Furthermore, protein consumed by participants was primarily a part of a mixed nutrient meal, rather than a pure protein meal typically prescribed in the laboratory setting [3,9,10]. Carbohydrate and fat consumed in these meals would slow the digestive process, and amino acid delivery to muscle cells. Any protein consumed in addition to the optimal 20–40 g dose for muscle protein synthesis in these mixed meals may be utilized for anabolic processes over the time course of digestion.

A protein intake of 2.3–3.1 g/kg of fat-free mass has been suggested to be the most protective against losses of lean tissue during energy restriction in lean resistance trained athletes [12]. A higher protein requirement may be justified for bodybuilders during competition preparation, as they perform resistance and cardiovascular training, reduce energy intake, and achieve a lean condition [13]. Therefore, the higher protein intake during the in-season to prevent loss of muscle mass in these participants may be justified.

During the in-season period, carbohydrate consumption was carefully timed around exercise. Glycogen is an important fuel substrate during resistance training [14], with glycogen depletion reported to reduce exercise performance [15]. Carbohydrate supplementation before and during resistance exercise improves performance of high volume, exhaustive exercise [16,17], a characteristic typical of bodybuilding training [2]. During in-season energy restriction, carbohydrate consumption following resistance training would assist in the replenishment of muscle glycogen, facilitating improved recovery and enhanced capacity to maintain training volume and intensity in subsequent sessions [18]. Bodybuilders commonly perform multiple training sessions in a single day during the in-season, typically an aerobic and a resistance training session [2], therefore post-exercise carbohydrate ingestion would be important for maintaining training consistency.

Study participants discussed using a weekly refeed day during the in-season period to boost training performance, provide a mental rest, and assist in body fat reductions. Intermittent energy restriction for weight loss has garnered significant recent clinical and research interest due to its hypothetical capacity to alleviate metabolic and behavioral adaptations associated with reduced energy intake. These adaptations include increased appetite associated with neuropeptide expression [19,20,21], reduced energy cost of physical activity [22], and hormonal effects that promote fat deposition and loss of lean mass [19,20]. Intermittent energy restriction, or metabolic rest periods, have been shown to achieve similar weight and fat loss as continuous energy restriction, despite a higher overall energy intake [21,22]. Animal studies have shown that acute energy restoration (<24 h) can attenuate, or even abolish the orexigenic neuropeptide expression resulting from energy restriction [23,24]. The short-term restoration of energy balance, particularly through increased carbohydrate ingestion, would also increase intramuscular glycogen stores allowing greater resistance exercise performance [25].

During the peak week, participants discussed the use of several strategies to assist in achieving a lean, vascular appearance. Carbohydrate loading, and fluid and sodium manipulation had all been used by participants, with varying success. Only one empirical study has directly assessed changes in muscle girth from carbohydrate loading, finding no significant changes in relaxed or tensed muscle girths following a three-day carbohydrate depletion and subsequent three-day carbohydrate load [26]. This suggests carbohydrate loading may not produce the desired increase in muscle volume. Fluid and sodium manipulation to enhance visual appearance has not been empirically studied, however the desired improvement in muscle size and definition may not be obtained. Manipulating fluid intake to cause dehydration will result in a loss of fluid from all compartments, not just subcutaneous tissue [27,28]. Muscle water content is reduced [27], which may reduce muscle volume, an undesirable outcome for a competitive bodybuilder. Additionally, plasma volume is decreased with dehydration [27]; the common practice of “pumping up” prior to posing on stage may be less effective in increasing muscle size due to the detrimental effects of reduced plasma volume on muscle blood flow and volume [13]. Similarly, the manipulations in sodium consumption will not change the volume of the intracellular or extracellular compartments, only modifying urinary sodium output [29].

In the weeks following competition, participants reported an increased energy intake from a wider variety of foods, often leading to significant weight regain. Daily energy intake in the first two days post-competition was approximately twice that of the four weeks pre-competition in female bodybuilders, with an increase in body mass of 3.9 kg in the three weeks after competition [30]. Similarly, an average weight regain of 5.9 kg was reported in a group of male bodybuilders, with 46% of these participants reporting binge-eating episodes in the days immediately following competing [31].

Supplement use, predominantly creatine and protein powders, was common amongst the bodybuilders interviewed, while “pre-workout” formulas had been trialled, with unwanted side effects commonly reported. Protein and creatine supplementation have been demonstrated to be effective for increasing lean mass and strength [32,33]. The efficacy of so-called “pre-workout” supplements is yet to be confirmed. These products contain a combination of key ingredients such as creatine, caffeine, arginine, β-alanine and selected plant extracts [13,34,35]. Efficacy would be dependent on the supplement ingredients, and some produce side effects such as acute increases in blood pressure and difficulty sleeping [34].

Bodybuilders have historically relied on magazines, other successful competitors, and more recently the internet, for information on dietary strategies [1]. This study identified the internet, in particular bodybuilding and strength and conditioning websites and forums, as a primary source of education, as well as other bodybuilders and coaches. In addition to the internet [36], athletes have previously identified family members, other athletes, coaches and registered dietitians as important sources of information regarding nutrition and dietary supplements [37,38,39]. Dietitians were not identified as sources of information by participants in this study, suggesting that their role needs better promotion amongst bodybuilders. With skills in dietary assessment, planning and body composition measurement, as well as evidence-based strategies demonstrated to assist in the accrual of lean mass, dietitians have much expertise to provide bodybuilders, particularly novices who were considered by participants in this study to be vulnerable to inappropriate strategies promoted on the internet.

Study limitations include use of a small, homogeneous sample. Experienced bodybuilders were purposively sampled, therefore these results may not reflect the wider bodybuilding population, particularly inexperienced bodybuilders.

## 5. Conclusions

Despite the common perception that bodybuilders follow extreme, unproven methods, the experienced bodybuilders in this study reported predominantly using dietary strategies recognized as evidence-based. However, inexperienced bodybuilders may be vulnerable to more extreme strategies based on advice widely disseminated on the internet and social media.

Novel strategies identified in this study warrant further investigation. Intermittent energy restriction, and hormonal responses associated with short-term energy restoration, should be studied to determine benefits for weight loss whilst maintaining lean mass in both lean-athletic and obese populations. Peak week strategies implemented by bodybuilders, such as fluid and sodium manipulation, require further investigation to determine their efficacy and safety.

## Figures and Tables

**Table 1:** Individual participant characteristics of seven experienced male, natural bodybuilders participating in in-depth interviews.

Participant	Age (Years)	Years of Bodybuilding	Number of Competitions	Competition Category	Level of Competition and Competition Success
Oliver	43	8	15	Masters; weight category	National (fourth place)
Luke	40	17	15	Opens; weight category	International (winner); Pro card
Kyle	25	7	15	Opens; weight category	International (winner); Pro card
Keith	22	7	8	Teenage; junior	National (winner)
Ben	30	13	12	Opens; weight category	National (fourth place)
Harry	32	10	9	Opens; weight category	State (winner); Pro card
Will	65	11	26	Grand masters; ultra-grand masters	International (winner)

Masters, >40 years; Teenage, <19 years; Junior, 19–22 years; Grand masters, >50 years; Ultra-grand masters, >60 years.

**Table 2:** Thematic summary of dietary practices and sources of dietary education, in seven experienced male, natural bodybuilders participating in in-depth interviews.

Themes	Subthemes	Counts of Coded Text	Indicative Quotes
Off-season	Meals	47	“Lunch would be, again, probably a 200-g chicken breast, one cooked cup of brown rice and maybe about 100 g of green veggies… Meal four, which is afternoon tea, which, prior to gym, is exactly the same as the meal before, the lunch meal, so the chicken, rice, veggie one, then after gym, which would be dinner, would be usually a meat, a red meat, so a steak, maybe a 200 g, you know, rump steak, another cooked cup of brown rice and some veggies, and that’s dinner.” (Oliver) “I have a dose of protein and carbohydrate with each meal…for protein I usually cycle between a few different sources. I use whey protein, and then of course the one that is salmon, white flesh fish, kangaroo and beef, they’re going to be my primary, I’ll cycle between those different protein sources” (Keith)
	Carbohydrates	6	“I dose my carbohydrate really high, because I want to make sure that my glucose metabolism is the best it possibly can be, because I will always diet on a high carbohydrate template to keep my training intensity high.” (Keith)
	Protein	3	“Anywhere from 2.2 to 2.9 g per kilo body weight. That’s not total lean mass but just my total body weight.” (Keith)
	Fat	3	“I will direct my fat anywhere from 0.5 to a maximum 1.2 g per kilo, so I keep my fats relatively moderate.” (Keith)
	Energy	3	“So I might sit at anywhere from, I used to sit at between 4500 and 5000 calories [per day] in my off-season.” (Keith)
In-season	Meals	34	“Each meal, just to start cutting the calories a little bit. The egg yolks would go from the eggs at night, just down to egg white, just, again, to start cutting some calories, and they would slowly go down, so in four eggs would go only three yolks. And then a couple of weeks later it’ll be down to two yolks and then one yolk.” (Luke)
	Carbohydrates	16	“The carb value will slowly come down. Around training, it’s going to remain quite high and in the morning it’s high-ish. But, yes, the carb value will slowly come down.” (Kyle) “Usually I make a drop, and I will either dig from fats, or carbs, or a combination of. I’m generally in favor of dropping carbohydrates initially and then digging into fats later,” (Keith) “I don’t have an issue with energy when I have my carbs around my training time, so pre-, intra- and post-workout is when I consume the majority of my carbohydrates through the day,” (Luke) “I will actually introduce more carbohydrate for fuel, you know, to fuel the requirement to get through, say, a 35-min interval session,” (Oliver)
	Protein	7	“I normally keep protein static. I’ll set it slightly higher than the off-season at the start of my prep and then just keep it the same throughout even if I lose weight. So if you were to look at it from a gram per kilogram basis, it would look like it’s going up, but it’s the same gram amount. So I’ll start at 225 g protein and just keep that throughout, so that will be roughly like 2.3, 2.4 g per kg,” (Harry)
	Fat	7	“I think I start with my fat probably around 25% [of energy] and then it might get as low as 15% to 20% at the end… So a day at the very end might be 40 g of fat.” (Harry) “So for example, I might start [the in-season] with my fat around 65 g [per day] and then that will only get decreased by maximum of 25 g while the carbohydrates can drop from, you know, 250 [g] at the start or 275 [g] all the way down to 100 [g] at the end on my low days,” (Harry)
	Energy	15	“So I probably start on average about 2400–2500 calories [per day] across the seven days, and I probably finish around 2000 or 1900 [per day] with probably a twofold increase in cardio.” (Harry)
Refeed days	Refeed days	32	“I have one day that’s closer to my, like my off-season calories. So that might be like 2800 calories on a day predominantly increasing carbohydrate. That’s to kind of stimulate further losses to prevent some of the downgrades in my energy expenditure you could say, and to replenish glycogen, to feel mentally refreshed, to get a break in.” (Harry)
Peak week	Carbohydrate loading	39	“So normally, I will increase my carbohydrates early in the week, sometime around Tuesday or Wednesday for Saturday show, taper them back down but not all the way down where they were at the lowest low. So maybe 400 [g] for a day and then down to say 350 [g/d], 300 [g/d], 250 [g/d], and then on Friday and Saturday, the show, I will be closer to 300 or the 400 [g/d] range to kind of fill back out. So it’s basically kind of like a modified carb loading strategy an endurance athlete would use.” (Harry) “The idea is to, you know, wring out the sponge, I suppose, of the last stage of leaning out in those depletion days, and they would be paired with high volume gym work, and the theory behind it was, apparently, to swell the muscle belly, it’s not a vascular thing, it was actually just increased overall fullness of the muscle once you flooded it with carbohydrate.” (Oliver) “He felt I looked my best, you know, 24 h prior to the competition, so all these little things you’ve sort of got to take note of and you think, all right, I look this good now, it’ll be even better tomorrow, and in my case it wasn’t, and you think, well, maybe we just do a carb load of two days next time around instead of three, if that works perfectly for that timeframe.” (Oliver)
	Water loading	17	“So then the water is still going in around about ten litres a day… then the water would start to, the water would start to cut back again as well and that was, sort of, you know, Thursday might still be up around about the ten litres, but then Friday and Saturday, Friday might cut down to around about four litres and then Saturday was two litres prior to, sort of, two o’clock or something like that… And then, you know, nothing, yes.” (Luke) “Muscle is 70% water and I’m not aware of any mechanism that tells the body to go after subcutaneous water. If you’re going to dehydrate, it’s going to be from everywhere and why are you pulling 70, you know, why are you pulling so much volume out of your muscles because you’re really wanting your muscles to be volumised?” (Will) “Those things don’t work for me,” (Ben) “A terrible, terrible thing to put your body through,” (Luke)
	Sodium manipulation	12	“So on the Monday, Tuesday, Wednesday would be salt in each meal, with probably around about two grams of salt, a gram, yes, one or two grams of salt with each meal, which was great, but then by Wednesday, oh man, you’ve just had this salty fishy chicken meal, it’s just absolutely disgusting and terrible. And then on the Thursday, Friday, Saturday, the salt would be dropped out.” (Luke) “It’s such a variable which can be really, really… Completely screw you up… Like, if you diet for 16 weeks and then the last two days you mess around with your sodium, and then you come on the stage bloated, it’s such a… It’s such a bummer.” (Kyle)
Post-competition	Post-competition	15	“You kind of work yourself up into a frenzy,” (Ben) “It’s not so much hunger, it’s more so flavour. It’s more sort of like I want a pizza because I haven’t had it in months,” (Kyle) “We eat everything we haven’t eaten all year,” (Will)
Supplements	Protein powders	23	“I take, obviously, protein powders. I take WPI [whey protein isolate] just because it’s, you know, it’s fast to absorb, or whatever… And then obviously, yes, and then obviously casein at night.” (Kyle)
	Creatine	15	“I don’t think I’ve stopped taking creatine monohydrate since 2004 to be honest.” (Harry) “The only thing I ever saw a result from was creatine. My wife would always say, ‘You’ve started using that creatine again, haven’t you?’ I’d say, ‘Why?’ She’d say, ‘Oh, you’ve got that swollen look about you, you know, that volumized look.’” (Will)
	Glutamine	10	“Glutamine is ten grams post-training in the off-season. Once I’m in diet mode for comp, especially the last four or five weeks, I up that to around about 40 g a day.” (Luke) “It’s supposed to help with your immune system and anticatabolic, so being on a lower calorie diet, I’m trying to stop muscle catabolism and glutamine is supposed to help out. And the last three times that I’ve dieted, I’ve, before that, the last four weeks I used to always get sick, always catch a cold or something. The last three times I’ve dieted, I’ve upped, had 40 g of glutamine a day for the last four or five weeks and I haven’t gotten sick.” (Luke)
	Preworkouts	9	“And it worked really well. It was, I was really focused in the gym… I just wanted to keep on training. I was just thinking about training, thinking about what I was doing at that time and was getting really into, into that workout.” (Luke) “I’m quite sensitive to caffeine by itself and I’ve had some of those preworkouts and not gotten to sleep until one or two o’clock in the morning and that’s having had it at 4:30 in the afternoon, five o’clock in the afternoon. So I’ve actually stayed away from those because of that.” (Luke)
Sources of education	Other bodybuilders	15	“He’s just been competing for, I don’t know, like, a lot of years, so, yes. He kind of, he is the guy who I’ll run everything by him. If I have an idea, like, should I do this maybe with my, you know, carbs, or whatever, I’ll run it by him first and he’ll give the okay or he’ll say, maybe just try this.” (Kyle) “They might have good body parts and, you know, if you get your legs looking like that or your back looking like that and you see what sport they’ve come from or what type of training they do for that body part, but then again, it may just come down to a genetic predisposition for that particular body part.” (Luke)
	Internet	15	“When I first got into it, I was not nearly as versed in the, I guess, the empirical evidence kind of way of thinking. I was reading posts online, bodybuilding.com forums. I was a regular on it.” (Harry) “Just Googling, you know, bodybuilding, you’ll get a… you will get some good information but you… they don’t necessarily know what is good and what’s bad.” (Harry) “The internet’s going to be everyone’s first port of call,” (Kyle) “The internet is littered with online gurus,” (Oliver) “It then just comes back to social media, and it’s the problem what I call the good-looking trainer. So the most popular ones with the most likes, whatever, let’s face it, they’re the good-looking blokes or the good-looking girls, most of which, unfortunately, don’t have that much between their ears but they have a huge following because most of their posts they’ve got their shirt off or they walk around in a bikini and everyone thinks they look great, so whatever they’re about to tell you must be good, rather than some rough-headed coach who’s in his 60s who’s done this sort of stuff all his life,” (Oliver) “He’s 17 years old and he’s following all these guys on Instagram and Facebook and things like that, and I don’t think they know. I’ve told him, ‘Mate, he’s not natural. Sure, have that as an attainable goal in your mind. If you fall short of that, you’re still going to be looking great.’ But I said, ‘Be under no illusion that that is natural,’ so I think a lot of the guys don’t know. They’re naive to it,” (Luke)
	Science and evidence-based sources	7	“I did very quickly gravitate towards more what I perceived to be more science-based and evidence-based approaches rather than just what were the big guys doing. To me, it was relatively intuitive that some genetic freak on a butt load of steroids and what worked for him would probably not be the same thing as what works for a more or less average bodybuilder who wasn’t going to be taking drugs.” (Harry)
	Coaches	6	“There’s not a whole lot of open information and sort of themes it’s just passed down from coaches in a tradition… I suppose I learn the majority of what I do through coaches and colleagues I worked with over time.” (Keith) “There are also a lot of 'coaches‘ out there who don’t, who are the same as them, you know. Most people, they compete in one or two shows and, you know, read a few magazine articles and they think they know how to be a coach. So the average coach is not a… the average coach doesn’t even have a bachelor degree to be honest.” (Harry)
